# A retrospective comparison of intraoperative CT and fluoroscopy evaluating radiation exposure in posterior spinal fusions for scoliosis

**DOI:** 10.1186/s13037-017-0142-0

**Published:** 2017-12-21

**Authors:** Jacob Riis, Rebecca R. Lehman, Robert A. Perera, John Ryan Quinn, Patricia Rinehart, Hans Robert Tuten, Victoria Kuester

**Affiliations:** 1 Department of Orthopaedic Surgery, Virginia Commonwealth University, 1201 East Main Street, Richmond, VA USA; 2 Department of Biostatistics, Virginia Commonwealth University, Richmond, VA USA; 3 Tuckahoe Orthopaedic Associates, Richmond, VA USA

## Abstract

**Background:**

Radiation exposure is a concern in the field of medicine. Deformity spine surgeons depend on modalities that have high exposure through scoliosis x-rays or computed tomography. The use of fluoroscopy has increased with the increased use of pedicle screws. Recently other 3-D imaging devices with navigation have also been brought onto the market to improve accuracy of screw placement. There is concern that because of the use of CT, the radiation dose to the patient is increased, however there is little literature that directly compares the amount of radiation using the 3-D devices to traditional fluoroscopy. Although we know intraoperative CT decreases the amount of radiation to the surgeon and operating room staff, there is limited comparison data for exposure to patients. Our study focused on a comparison of radiation exposure data for pediatric scoliosis patients receiving posterior spinal fusions using traditional fluoroscopy and the Medtronic O-arm in an effort to determine the method most likely to decrease radiation exposure in the pediatric population.

**Methods:**

Retrospective review of data in patient charts from two pediatric surgeons practicing in both a University and private hospital setting. Data collected included age, weight, height, diagnosis, Cobb angle, fusion levels, number of screws, and number of hooks, O-arm spins, fluoro doses and O-arm doses. Effective dose was calculated using output measures and radiation doses were compared along a continuum that took into account the amount of correction as indicated by Cobb angle.

**Results:**

A total of 57 patients, 25 using the O-arm and 32 using traditional fluoroscopy, were analyzed. Effective dose was calculated and then compared as a factor correlated to curve severity. At lower angles of correction we found no statistically significant difference between methods in terms of effective radiation dose. There was no statistically significant divergence until a Cobb angle correction of greater than 74 degrees, where the Oarm dose was shown to be lower by comparison.

**Conclusion:**

We found that regardless of the methods used there is still a significant radiation dose that is utilized in scoliosis procedures. The two methods analyzed did not display statistically significant differences in effective dose for the average case. Safely managing radiation exposure for pediatric patients is of the utmost priority. Healthcare professionals, however, face repeated exposure to radiation over the course of a long career. In our data set the O-Arm system does not increase overall exposure for patients and decreases radiation doses for providers and thereby provides a safe alternative to traditional fluoroscopy without compromising accuracy of implant placement or patient care.

Level of Evidence: III.

## Background

Radiation safety is an ever present concern in the field of orthopaedics. Spine surgeons, in particular, have a vested interest in the safe and appropriate use of radiation due to the nature of their practice [1]. Improvements in spine stabilization systems, increased use of pedicle screws and advances in imaging technology have driven a renewed interest in evaluating the manner in which we approach the issue of radiation safety [2–5]. There are several factors to consider when evaluating an imaging method to be used in the clinical setting. The primary area of concern is that of the wellbeing of the patient. As we endeavor to improve the clinical outcomes, we simultaneously must provide care in a safe, cost efficient manner. Radiation doses also have to be evaluated in the context of lifetime risk to the pediatric population [6, 7]. A second consideration is the safety of the provider [8]. Spine surgeons may have an awareness of the radiation risks of their profession, but the amount of exposure, cumulative exposure and long term effects are often underappreciated [5, 9]. New advances in imaging technologies have brought additional variables to bear on the question of patient and provider safety [10]. Several studies in the past 5 years have compared intraoperative navigation techniques with traditional techniques in terms of effective dose, but there are a limited number of studies that directly compare a surgeon using the O-arm exclusively with a surgeon who primarily uses a C-arm [2, 11]. In general the current literature suggests that CT guided pedicle screw placement results in higher doses of radiation for the patient and lower doses for providers [1, 8, 10–14]. Our purposed study focused on evaluating two of the most common radiographic techniques for pedicle screw placement in scoliosis surgery, the traditional fluoroscope and Medtronic O-Arm (Medtronic,™, 710 Medtronic Parkway Minneapolis, Minnesota, 55,432–5604). We evaluated these methods that were part of a standard practice to answer one basic question: Is there a measurable effective dose difference between the two methods for pedicle screw placement in pediatric scoliosis surgery? In addition to this basic question which has been addressed to some degree in the current literature, we sought to take into consideration the severity of the corrected curvature of the spine as an additional variable. Our study took calculated effective doses from both methods at the time of surgery and subsequently compared the doses to each other rather than to a historical value. Our hypothesis, therefore, was that there was a significant detectable difference between the two methods with respect to radiation dose. Statistical analysis also was utilized to compare effective doses along a continuum that took Cobb angle into consideration. While this information will not answer all questions regarding outcomes of surgery, accuracy of screw placement, long term results of radiation exposure, or provider biases due to training or experience, it can provide a foundational perspective upon which future research can be based and an additional point of discussion regarding safety that can be added to the conversation with patients regarding the chosen method.

## Methods

An IRB was submitted and reviewed by the IRB committees of both Virginia Commonwealth University Hospital and St. Mary’s Hospital and approved on 1/17 2014 as exempted research because of no greater than minimal risk to the children and an exemption for consent and assent as outlined in 45 CFR 46.404.

**Fig. 1:**
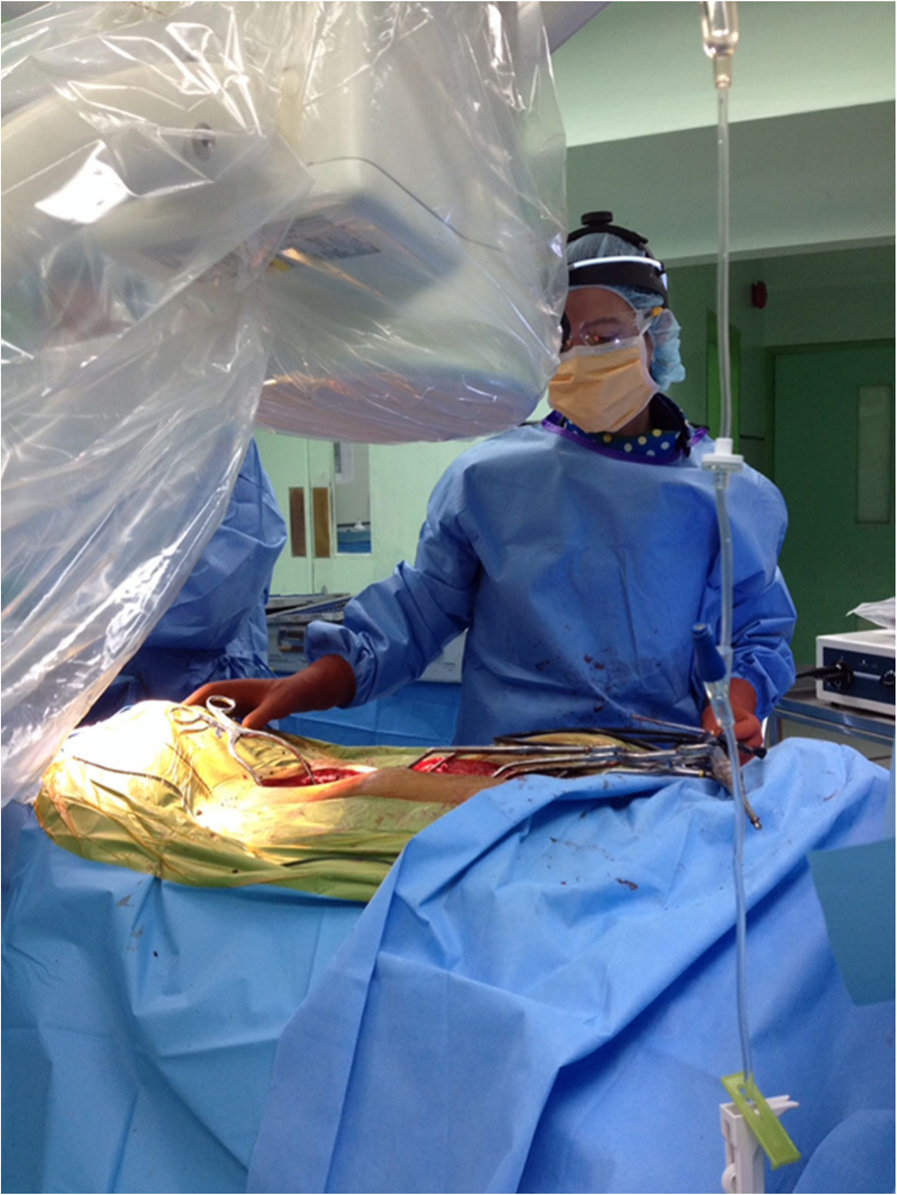
Typical Flouro positioning and setup

**Fig. 2:**
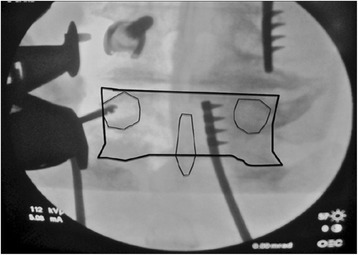
Intraop imaging, c-arm

**Fig. 3:**
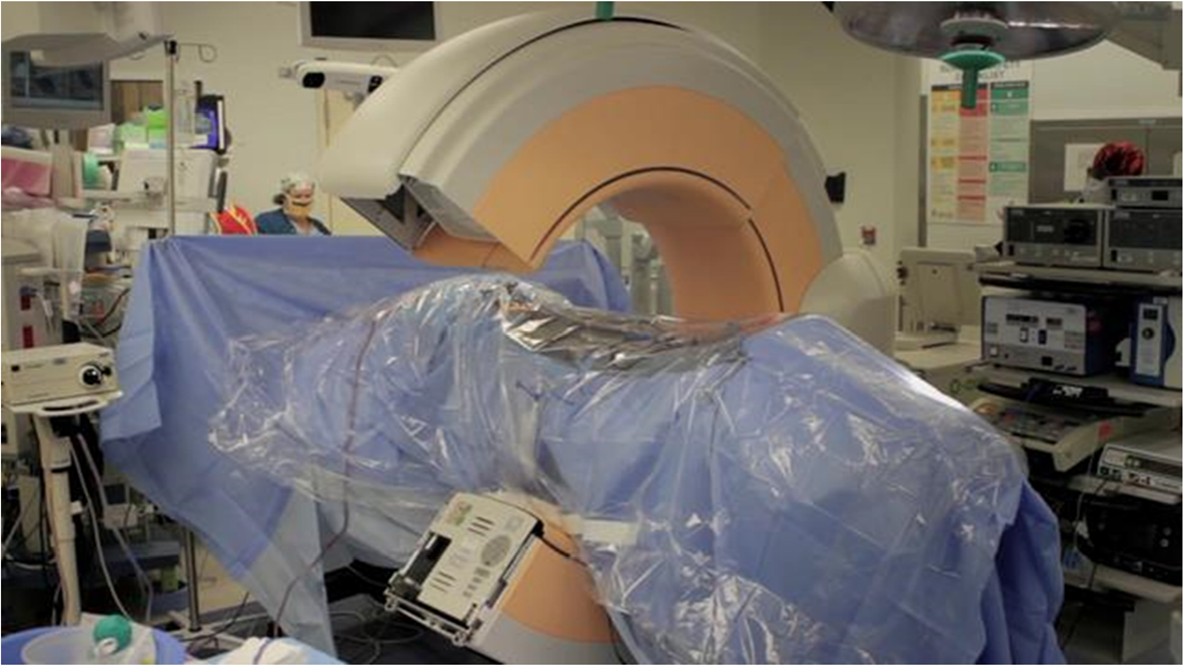
Typical intraoperative CT setup

**Fig. 4:**
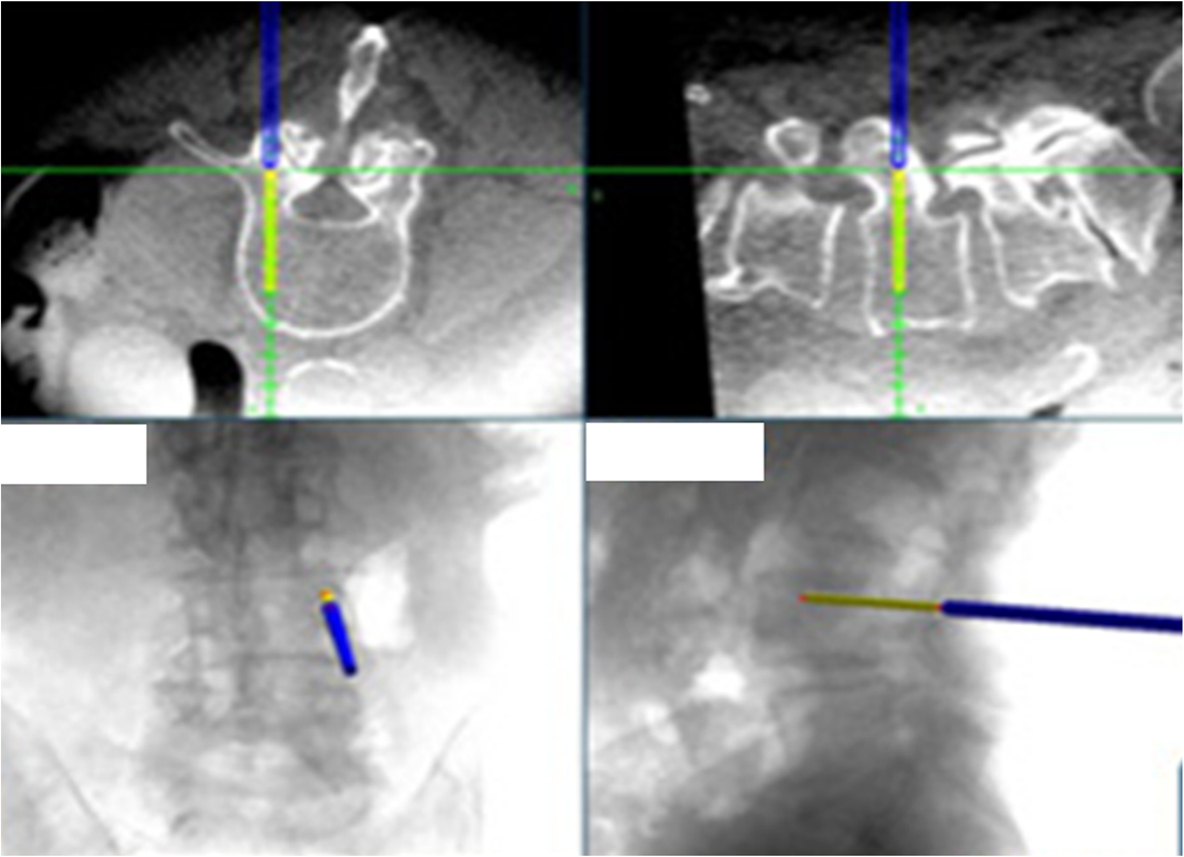
Typical intraoperative CT guidance images

All scoliosis cases performed by the surgeons identified in the collection period were evaluated. All providers used techniques that they had been using as part of routine practice and no techniques were experimental or deviated from FDA approved application of technology (Figs. 1, 2, 3 and 4). Data collected includes age, weight, height, primary diagnosis, Cobb angle, fusion levels, number of screws, and number of hooks, O-arm spins, fluoroscopic doses and Intraoperative CT doses. Cobb angle analysis was performed by experienced pediatric orthopedic spine surgeons (RT and VK). Radiation doses were verified by two radiologists and conversion of radiation doses to effective dose (Msev) was assessed by research team with assistance from a radiation physicist. Effective dose calculations for fluorosocopy were completed using variables obtained using the Monte Carlo method of dosimetric calculation using a mathematical phantom. The Monte Carlo method is a numerical system used to solve equations or calculate integrals based on random number sampling [11, 13]. It is typically used in settings were the number of variables and the interplay between variables is highly complex and allows for reproducible generalizations that correlate to measured findings. In the setting of dosimetric calculations it utilizes established parameters to generate factors that can be used with raw data to generate standardize dose equivalents. Due to the inherent complexity of measuring radiation doses, Monte Carlo algorithms have been developed and integrated into proprietary software that utilizes static factors and then generates coeffiecients that can be used to translate raw exposure data(mGy) into effective dose. In the setting of a computed tomography scan(CT) this is simplified by a fixed gantry distance, fixed incident angles and apetures, and fixed detector distance [15–17]. Effective dose calculations for CT are generally well established and on newer models are often calculated at time of imaging. Fluoroscopy, on the other hand, with it’s variable angles, adjustable distances between detector and patient, readily modified kVp and mA, presents challenges that require some assumptions for a ‘best fit’ calculation. General factors that were standardized included distance from detector, air kerma, incident angle, and body weight. Tissue weighting factors were then utilized and an average weighting factor was used to calculate effective dose based on incident radiation (mGy) for fluoroscopy and the dose length product for the intraoperative CT. Statistical evaluation of the effective dose was then undertaken and evaluated mean effective doses, dose per implant calculations, and comparative evaluation of severity of curve. Outcome measures were focused on effective dose as it related to curve severity and imaging methodology. We hypothesized that there would be a significant difference in radiation doses in more severe curves for all techniques. Patient demographics are summarized in Table 1.

**Table 1:** Demographics of patients with associated diagnoses. Disease profile

	Fluoroscopy	Intraop CT
Diagnosis		
Adolecent Idiopathic Scoliosis	22	23
Spina Bifida	2	1
Congenital Scoliosis	1	1
Neuromuscular D/o	6	1
Other	2	0

### Statistical methods

Summary statistics (mean, standard deviation, 95% confidence interval and sample size) were reported for all variables by imaging type. It was of interest to analyze the difference in patient exposure to radiation measured in Msev between the intraop CT and fluoroscopy imaging types while taking into account for the severity of the curve. While this does not take into account the patient comorbidities, cause of scoliosis or overall operative time, it does serve as a proxy for the relative amount of correction and allowed for greater statistical power by comparing variables in a continuous manner as opposed to artificially subdividing into smaller subsets. Two participants, one from each imaging type, were removed from the analysis due to missing data on the Cobb angle bringing to the total sample size analyzed to *N* = 57. An ANCOHET model, an extension of the ANCOVA allowing for heterogeneity of regression was used for the analysis [18, 19]. The model took into consideration imaging type, Cobb angle and the interaction between imaging type and Cobb angle as covariates. The distribution of the residuals was examined for normality using QQ plots. To determine whether there is any statistically significant difference in radiation exposure between the two imaging types, follow up tests were performed testing for differences in the predicted Msev between the two imaging types at varying Cobb angles. To account for multiple testing, a comparison procedure was used to control the experimentwise type I error rate. This correction for simultaneous tests allows for any number of Msev values to be compared while controlling experimentwise alpha to the desired level of *p* < 0.05. Cobb angles were examined along a continuum to determine if at any degree of Cobb angle the difference between imaging types becomes statistically significant. R statistical software version 3.1.2 was used for all calculations.

## Results

**Fig. 5:**
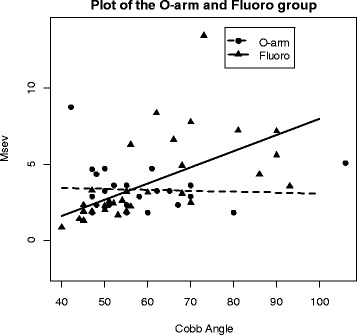
Scatter Plot of effective dose plotted vs cobb angle. Plot of the data for both the O-arm and Fluoro group. It depicts the linear regression lines for each and exhibits the different slope values

Height, weight, age, Cobb angle and Msev are summarized with means, standard deviations, 95% confidence intervals and sample sizes by imaging type: Introperative CT (*N* = 26) and Fluoro (*N* = 33) in Table 2. Before analysis of the data, two of the observations were removed due to missing data on their Cobb Angle. Final analysis included 25 patients from the O-arm group and 32 from the Fluoro group. The residuals were reasonably normally distributed and homoscedastic. The results from the ANCOHET produced a significant main effect of Cobb angle (*F*_1, 54_ = 6.89, *p* = 0.011) and a significant interaction between the Cobb angle and imaging type (*F*_1, 54_ = 8.86, *p* = 0.004). No significant main effect of imaging type was found (*F*_1, 54_ = 0.38, *p* = 0.541). A significant interaction indicates that the effect of imaging type depends on Cobb angle. Figure 5 shows the observed data plotted against their predicted based on Cobb angle. A significant interaction prevents interpretation of main effects so predicted Msev values were compared across the Cobb angle. After correcting for multiple testing we found that there was a statistically significant difference between the two imaging types at Cobb angles 74 (${t}_{53}^2$ = 6.24) and above and no statistically significant difference below a Cobb angle of 74. Some caution should be taken in interpreting this data given the limited data at large Cobb angles.

**Table 2:** Summary statistics for variables: height, weight, age, Cobb Angle and Msev by imaging type

	Intraop CT			Fluoro		
	Mean (SD)	95% confidence interval	N	Mean (SD)	95% confidence interval	N
Height	160.2 (8.89)	(156.6, 163.8)	26	158.9 (14.26)	(153.8, 164.0)	33
Weight	55.2 (13.39)	(49.8, 60.6)	26	54.2 (17.55)	(48.0, 60.4)	33
Age	14.5 (2.40)	(13.5, 15.5)	26	16.9 (9.85)	(13.4, 20.4)	33
Cobb Angle	58.2 (13.47)	(52.6, 63.8)	25	60.1 (15.08)	(54.7, 65.5)	32
Msev	3.3 (1.51)	(2.7, 3.9)	26	3.7 (2.69)	(2.7, 4.7)	33

## Discussion

Radiation exposure is a critical element for both the provider and patient in complex spinal surgery. There has been a renewed interest in recent years to addressing the risks of radiation in newer forms of intraoperative guidance for spine surgery [1, 2, 8, 10, 11, 13, 14]. In our analysis we found a statistically significant difference in radiation dose that favored the use of the intraoperative CT at Cobb angle values greater than 74 degrees. Below 74 degrees there was not a statistically significant difference between methods based on effective dose calculations. The primary purpose of our study was focused on the radiation dose to the patient. It is important to note, however, that consideration of dose to the provider is an added factor. The traditional fluoroscopic techniques subject the surgeon to levels similar to the patient, but on a frequent basis. This risk is mitigated primarily through prevention, physical barriers and monitoring. One of the singular advantages of intraoperative CT is that it does not subject providers any additional radiation. The risk over a lifetime of providing surgery could then be theoretically reduced significantly using this technique. An additional factor that was not a studied variable in this study was relative experience of the provider. An experienced provider would likely need fewer fluoroscopic views to accurately place pedicle screws and might mitigate the risk to both the patient and provider. While intraoperative CT may level the playing field it relies heavily on technology that has to be both understood and used properly to derive its full benefit. Dosing patients up front with large doses of radiation could pose problems if software proved unreliable or if verification was needed and additional spins were required.

## Conclusion

We concluded that there was no significant difference in dose between radiographic methods in the hands of providers using their preferred method for the majority of scoliosis correcting procedures in our data set. Further, we found that in the most severe curves there was likely some benefit in the use of the intraoperative CT over fluoroscopy in total dose. Further prospective, appropriately powered, randomized research will be needed to determine if this effect is limited in its scope or generalizable to pediatric spine surgery practices.
